# The role of iron in normal and impaired testicular function

**DOI:** 10.1111/andr.70068

**Published:** 2025-06-04

**Authors:** Aileen Harrer, Esther G. Meyron‐Holtz, Andreas Meinhardt

**Affiliations:** ^1^ Unit of Reproductive Biology Institute of Anatomy and Cell Biology Justus Liebig University of Giessen Giessen Germany; ^2^ Hessian Centre of Reproductive Medicine Justus Liebig University Giessen Giessen Germany; ^3^ Faculty of Biotechnology and Food Engineering Technion ‐ Israel Institute of Technology Haifa Israel

**Keywords:** ferritin, ferroptosis, iron, IRPs, testicular infection, testis

## Abstract

Iron plays a critical role in testicular physiology, impacting spermatogenesis, testosterone production, and overall testicular function. Iron homeostasis is maintained through systemic and cellular regulatory mechanisms, including hepcidin‐mediated systemic iron control and the iron‐responsive element/iron regulatory protein (IRE/IRP) system at the cellular level. Germ cells require iron for cell divisions and development, while Leydig and Sertoli cells depend on iron for the biosynthesis of testosterone and the maintenance of sperm development, with iron‐dependent enzymes playing essential roles in lipid metabolism, DNA synthesis, and mitochondrial function. Iron imbalances, including iron overload and deficiency, significantly impact testicular health. Excess iron accumulation induces oxidative stress, lipid peroxidation, and ferroptosis, contributing to testicular dysfunction. Hereditary hemochromatosis and beta‐thalassemia major are associated with disrupted iron metabolism, leading to altered hormonal profiles and impaired spermatogenesis. Conversely, iron deficiency, often linked to dietary insufficiency or inflammatory conditions, can impair testosterone production and sperm quality. Bacterial and viral infections of the testis, such as those caused by *Escherichia coli*, Zika virus, and HIV, also alter iron metabolism, influencing immune responses and exacerbating testicular inflammation. Additionally, chronic conditions such as obesity and diabetes may disrupt iron regulation and contribute to reproductive dysfunction. Emerging evidence suggests a strong link between ferroptosis and testicular damage, with iron‐dependent lipid peroxidation playing a key role in cell death pathways. This review highlights the complexity of iron regulation in the testis, the negative effects of disruptions of iron metabolism on male fertility and reproductive health as well as the gaps in knowledge that currently prevent therapeutic approaches to mitigate infertility associated with iron dysregulation.

## 1 IRON HOMEOSTASIS

### 1.1 Iron

In humans about 2% of all proteins are iron proteins that are either heme‐containing proteins, iron ion‐binding proteins, or iron‐sulfur cluster proteins. To secure continuous iron supply and at the same time protect cells from iron overload, systems that are involved in the cellular iron import, export, and storage of iron, are tightly regulated. This regulation takes place on a systemic level through the peptide hormone hepcidin and on the cellular level by several systems including the “iron‐responsive element/iron regulatory proteins” (IRE/IRP) system. The cellular compartments that are especially enriched in iron proteins are the mitochondria, the nucleus, and the endoplasmic reticulum (ER). Systemically, about 70% of iron is located in red blood cells. In recent years some progress has been made in understanding how the different regulatory systems are fine‐tuned to optimize iron distribution when iron is sparse. On the other hand, iron overload, which can cause oxidative damage including an increase in lipid peroxidation of polyunsaturated fatty acids was shown to be one of the mediators of ferroptosis, a form of regulated cell death.^1^, ^2^, ^3^, ^4^, ^5^

### 1.2 Iron transport and storage

Iron import to cells is initiated by receptor‐mediated endocytosis via transferrin receptor (TFR)1 (Figure 1). TFR1 binds to the plasma protein transferrin (TF), which, when iron‐loaded, has a high affinity to TFR1 at physiologic pH. Once the TF‐TFR1 complex is internalized and iron is released to the endosomal lumen the complex is recycled to the plasma membrane and the apo‐TF is released. The endosomal ferric iron (Fe^3+^) is reduced and transported to the cytosol through the divalent metal transporter (DMT)1. In selected cell types such as intestinal epithelium, DMT1 is the main importer of iron through the apical plasma membrane and additional mechanisms for cellular iron uptake have been described.^4^ In the cytosol, ferrous iron (Fe^2+^) is transiently bound to glutathione and possibly other small molecules of the intermediary metabolism. This transiently bound iron or labile iron pool (LIP) is the main iron source for the different iron proteins, for iron‐sulfur cluster assembly, and for heme production. The poly(rC) binding proteins (PCBP)1 and PCBP2 play important roles in chaperoning the iron ions to their target proteins in the cytosol and possibly also in the nucleus.^6^, ^7^ Excess iron can be stored in the iron storage protein ferritin. Alternatively, iron can be exported from the cell through the ferrous iron exporter ferroportin (FPN).

**FIGURE 1:**
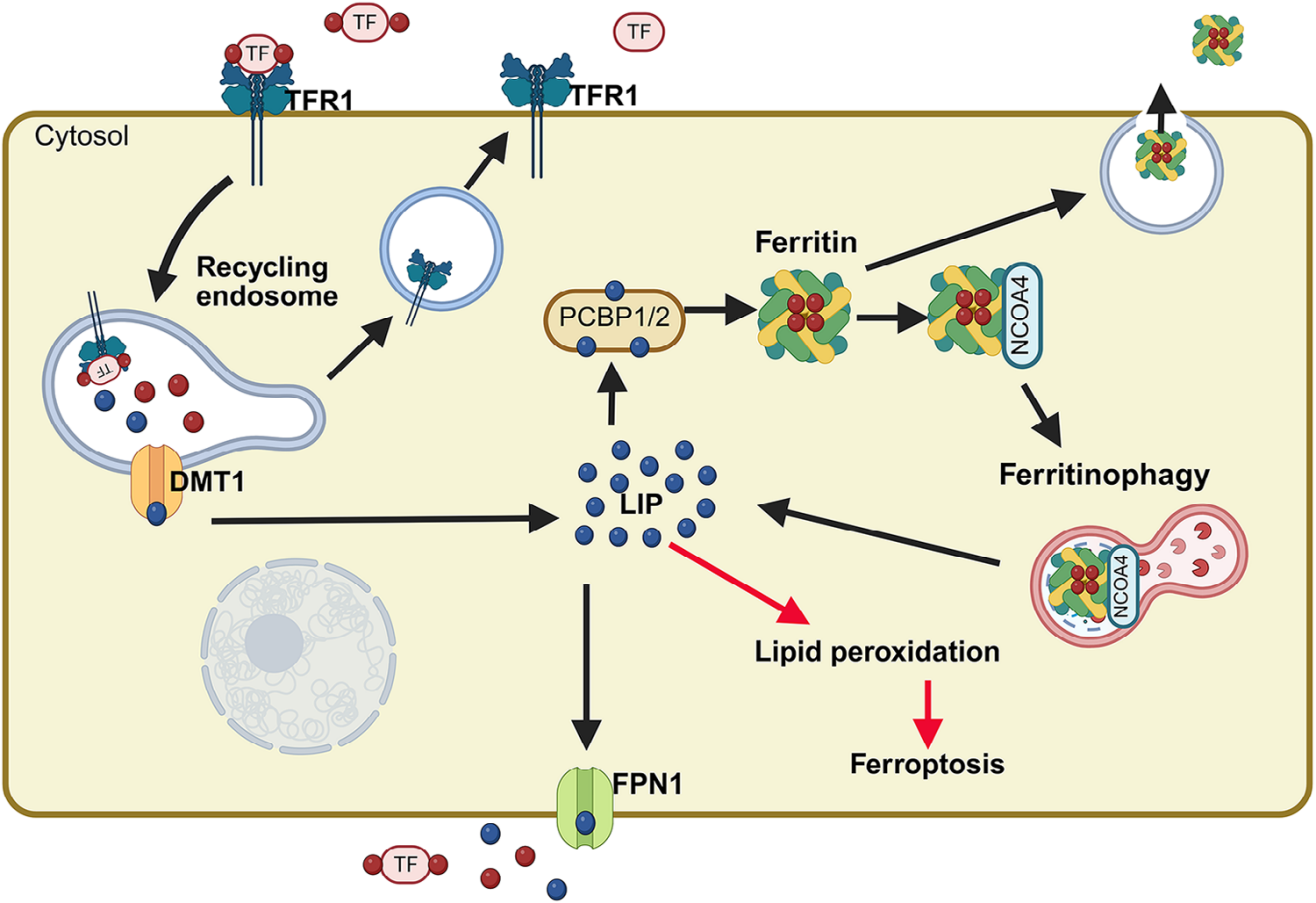
Simplified scheme of iron transport and storage. Ferric iron (Fe^3^⁺, red dots) is bound to transferrin (TF), which circulates through the bloodstream. The uptake of transferrin into the cells occurs via an endocytic process involving the transferrin receptor (TFR1), which is recycled and transported back to the membrane. In the endosome, ferric iron is reduced and transported via the divalent metal transporter (DMT1) into the cytosol. In the cytosol, ferrous iron (Fe^2^⁺, blue dots) is transiently located in the labile iron pool (LIP) or alternatively exported through the ferrous iron exporter ferroportin (FPN) or through the secretion of ferritin. The chaperone protein poly(rC) binding protein (PCBP) 1 takes Fe^2^⁺ from the LIP and transports it to the iron storage protein ferritin. Ferritin shuttles the iron to other cellular compartments, such as lysosomes. The entry of ferritin to the lysosomes (ferritinophagy) is facilitated by the nuclear receptor coactivator (NCOA)4. After entering the lysosome, ferritin undergoes degradation to release iron that is reduced in the lysosome and released to the cytosolic LIP. All these processes of iron transport are tightly regulated to maintain iron homeostasis. Too high cellular iron concentration can initiate an iron‐dependent cell death, ferroptosis (created with biorender.com/t2qwiqm).

Ferritin is a large protein nanocage built of 24 subunits of two kinds the L‐ and H‐subunits (FTL and FTH). FTH has ferroxidase activity and upon entry through one of the molecule's three‐fold symmetry pores, Fe^2+^ is oxidized and Fe^3+^ sequestered, building an iron core within the ferritin cavity that can contain up to 4500 iron ions.^8^ The assembly of ferritin and its iron acquisition are known to happen in the cytosol, yet ferritin also traffics between cellular compartments. To release its stored iron when cellular iron is sparse, ferritin must undergo degradation in the lysosome. About a decade ago, the nuclear receptor coactivator (NCOA)4 was recognized for its function as a ferritin cargo receptor, mediating the entry of ferritin into autophagosomes (ferritinophagy) and eventually into lysosomes.^9^, ^10^, ^11^ NCOA4 was also shown to play a role in the export of iron‐loaded ferritin by extracellular vesicles.^12^ In addition, we and others have demonstrated both, direct and vesicle‐mediated mediated export mechanisms for ferritin.^13^, ^14^, ^15^

Taken together, iron can be exported from cells through FPN and ferritin, yet ferritin‐iron export cannot compensate for the absence of functional FPN.^16^ One way to explain this may be through the different functions of iron secretion. While FPN‐secreted iron is mainly loaded onto TF and from there is distributed systemically, ferritin is most likely taken up by neighboring cells. We suggest this, based on the fact that ferritin that reaches the plasma, hardly contains iron, and also its subunit composition does not resemble the subunit composition of cellular or secreted ferritin (from cells in cell culture), suggesting that most of the ferritin secreted by cells never reaches the bloodstream.^17^

### 1.3 Cellular and systemic iron regulation

To ensure optimal cellular and systemic iron levels, intricate regulatory systems are required (Figure 2). The processes of iron acquisition, storage, and efflux must be precisely orchestrated to achieve and maintain iron homeostasis. At the systemic level, the primary regulator of iron homeostasis is hepcidin, a protein derived from the liver. Hepcidin exerts its control by blocking and inducing the internalization and degradation of the iron exporter FPN, thus affecting iron recycling from macrophages and intestinal absorption in the duodenum.^18^

**FIGURE 2:**
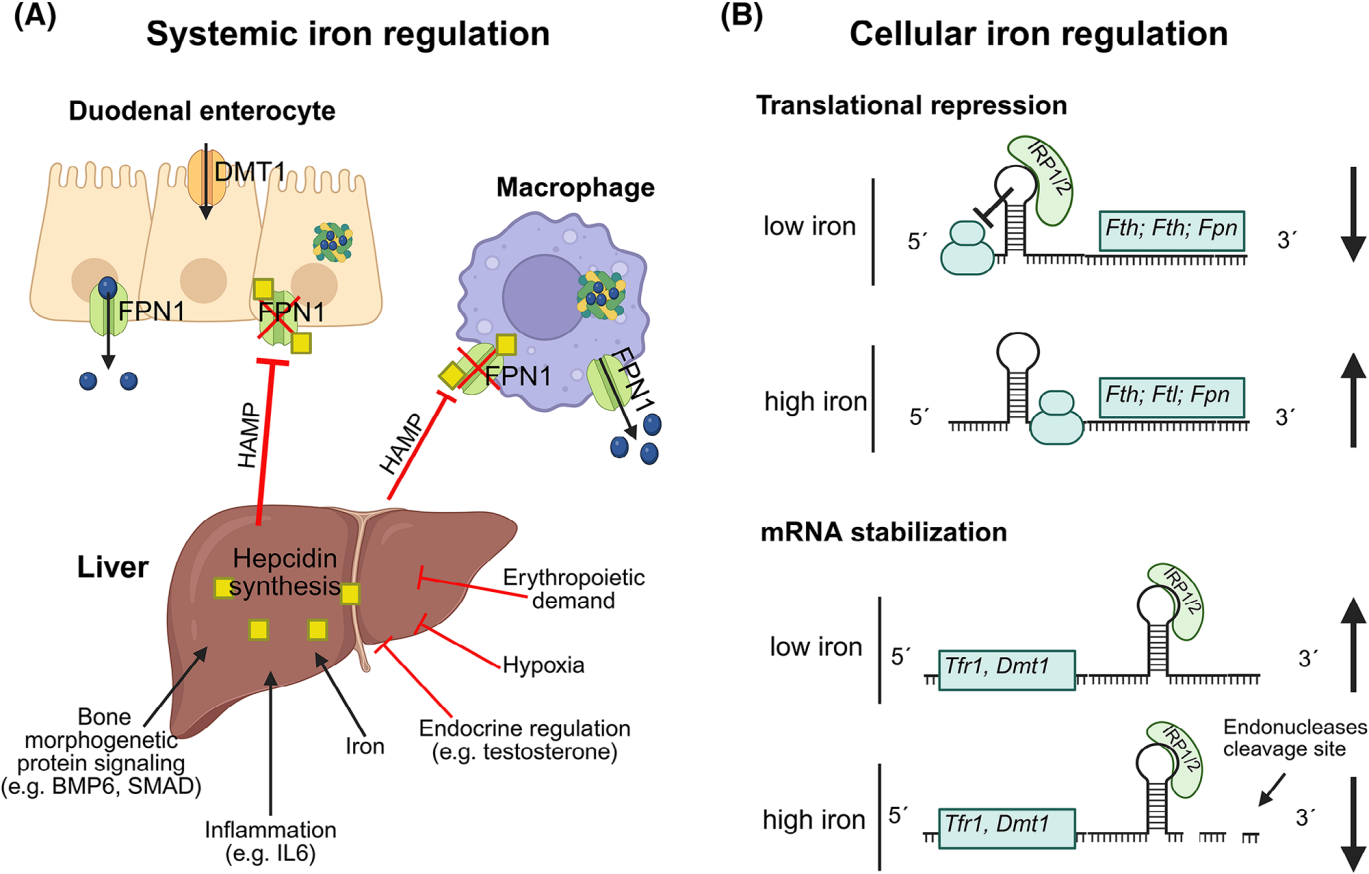
Scheme depicting the regulation of iron levels on a systemic and cellular basis by hepcidin, IRP1, and IRP2. Iron homeostasis is tightly regulated at both the (A) systemic and (B) cellular level. (A) For systemic iron regulation, the liver plays a central role, where hepcidin (HAMP) synthesis takes place. Hepcidin is synthesized in response to inflammation or high circulating iron concentrations to prevent the release of iron through ferroportin (FPN) in cells such as macrophages or duodenal enterocytes. When circulating iron concentrations are low, hepcidin synthesis is low as well, allowing ferroportin to release iron from the cell to recruit iron from the diet or internal iron cycles such as macrophage iron from erythrophagocytosis. (B) At the cellular level, the iron regulatory proteins (IRP) 1 and 2 play an important role in cellular iron regulation. Both proteins sense the cytosolic iron concentration within the cell and bind to specific stem loops when iron is sparse. This leads, for example, in the case of low iron concentrations in the cells, to inhibition of the translation of ferritin (*Fth/Ftl*) and ferroportin (*Fpn*), while the mRNAs for transferrin receptor (*Tfr*1) and divalent metal transporter (*Dmt*1) are stabilized for translation. In the case of high iron concentrations in the cells, the process is reversed: the storage proteins are translated, and the mRNA for *Tfr*1 and *Dmt*1 is degraded (created with biorender.com/t2qwiqm).

#### 1.3.1 IRE/IRP system

At the cellular level, the LIP is intricately controlled by the IRE/IRP system. The two cytosolic RNA‐binding proteins, iron regulatory protein (IRP)1 and IRP2 regulate cellular iron homeostasis through their binding to mRNA stem‐loop structures, (iron‐responsive elements) IREs, located in the 5′ or 3′ UTR of regulated transcripts. The IRPs control numerous proteins involved in iron regulation, such as TFR1 and the FTL and FTH subunits of ferritin. However, the ability of each IRP to recognize an exclusive subset of IREs suggests that IRP1 and IRP2 may each regulate unique mRNA targets, possibly extending their function beyond the regulation of intracellular iron homeostasis.^19^, ^20^ Both IRPs sense the LIP status, and their RNA‐binding activity is elevated when cellular iron levels are low. Yet, each IRP senses the LIP by different mechanisms.^21^ IRP1 is a bifunctional protein that acts as a cytosolic aconitase (cAco) that isomerizes citrate to isocitrate when it contains an Fe‐S cluster, and as an RNA‐binding protein (IRP1) when it is lacking the cluster.^22^ The ability of the protein to switch from cAco to IRP1 in low iron conditions depends on the stability of the Fe‐S cluster, which is sensitive to cellular oxygen and reactive oxygen and nitrogen species (ROS/RNS).^23^ Typically, around 95% of IRP1 is in the enzymatic cAco form, as the Fe‐S cluster of IRP1 is stable at physiologic oxygen levels.^24^, ^25^ In contrast, IRP2 is ubiquitinated and degraded by the proteasome in response to high LIP, oxygen, and ROS/RNS levels.^26^ Taken together, both IRPs are sensitive to cellular iron and oxygen concentrations, but in different ways. Both their RNA‐binding activities are increased by low cellular iron levels, but IRP2, responds to increased oxygen and ROS/RNS levels with increased degradation, while IRP1 requires high oxygen/ROS/RNS concentrations to undergo the conformational change from cAco to its RNA‐binding form.^27^

#### 1.3.2 Ferritin trafficking

An additional and important contributor to the regulation of cellular iron homeostasis is the internal cycle of ferritin. While ferritin reduces the LIP upon its biosynthesis by oxidizing and storing LIP‐iron in its cavity, it can also restore the LIP under low iron conditions, through the release of its stored iron.^28^, ^29^ The ferritin biosynthesis is regulated at the transcriptional level by the nuclear factor erythroid 2‐related factor (NRF)2 and at the translational level by the IRE/IRP system.^30^, ^31^ In addition, NCOA4, which mediates ferritinophagy, is regulated by iron and facilitates ferritinophagy when the iron is low. NCOA4 is mostly intrinsically disordered and participates in the formation of condensates with and without ferritin, facilitating macro‐ and microferritinophagy.^32^

#### 1.3.3 NRF2

A transcription factor that senses cellular oxidative stress, is constitutively expressed and rapidly degraded by the proteasome under homeostatic conditions.^33^ Increased oxidative stress mediates the dissociation of NRF2 from the complex that is responsible for its ubiquitination and degradation. Once released from this complex, NRF2 translocates to the nucleus where it has DNA binding sites and regulates over 200 genes. Many of these genes are involved in antioxidant defense and redox balance and some are important for iron homeostasis, including heme‐oxygenase1, both subunits of ferritin, FPN, and bone morphogenetic protein (BMP) 6, which takes part in the induction of hepcidin in the liver.^34^

#### 1.3.4 Hypoxia‐inducible factor

On the other end of the regulation of the oxygen spectrum is the hypoxia‐inducible factor (HIF), a transcription factor that senses and is activated by low oxygen levels.^35^ HIF consists of a stable HIFß subunit that forms a complex with one of the labile HIF1α or HIF2α subunits in the nucleus and functions as a transcription factor when in the complex. Similar to NRF2, the HIFα subunits are also constitutively expressed and are degraded by the proteasomal system in the cytosol when they are marked for ubiquitination by the iron‐dependent prolyl hydroxylases.^34^, ^36^ When the HIFα subunits are stable, because of hypoxia, iron deficiency, or other signals, they enter the nucleus, where they form the with HIFß and active DNA‐binding complex. HIF2α plays an important role in the regulation of iron homeostasis, as it controls dietary iron uptake in intestinal epithelial cells.^37^ Additionally, it regulates erythropoiesis, the most iron‐consuming pathway in the body. In the erythropoietin‐producing interstitial fibroblasts in the kidney, called Norn cells,^38^ HIF2α regulates erythropoietin production. HIF2α has an IRE in its 5′UTR which is regulated predominantly by IRP1, integrating iron and oxygen regulation in a fine‐tuned system.^20^

## 2 ROLE OF IRON IN NORMAL TESTICULAR FUNCTION

Testosterone production and spermatogenesis are the primary functions of the testis. Spermatogenesis occurs within the seminiferous tubules and is supported by Sertoli cells inside the seminiferous tubules, as well as by nutrients and hormones supplied from the testicular interstitium, which surrounds the seminiferous tubules. The interstitium harbors capillaries, lymphatic vessels, various immune cells, Leydig cells, and other cell types, all of which contribute differentially to the normal development of spermatozoa.

### 2.1 Testosterone biosynthesis

Leydig cells are the main producers of androgens, particularly testosterone.^39^ Testosterone diffuses into Sertoli cells, where it binds to androgen receptors, triggering gene expression essential for complete spermatogenesis. Beyond its role in reproduction, testosterone also regulates secondary male characteristics such as skeletal muscle growth and male hair pattern. The biosynthesis of testosterone is iron‐dependent. The initial steps of its biosynthesis from cholesterol are mediated by proteins of the P450 family. Both, CYP11A1, the enzyme that catalyzes the first step by converting cholesterol to pregnenolone, and CYP17A1, which facilitates the next two steps, are heme proteins.^40^ Both in vivo and in vitro studies have demonstrated that iron overload impairs testosterone biosynthesis and suggested that this process is especially sensitive to oxidative stress, mediated by iron overload.^41^, ^42^ Moreover, studies in iron‐deficient rats have shown a significant decrease in serum testosterone levels, despite unchanged serum cholesterol levels, and iron supplementation was able to reverse this effect. Because peroxidases and catalase are also heme proteins, it has been suggested that iron deficiency may disrupt testosterone synthesis by reducing protection against oxidative stress. Taken together, iron plays a crucial role in testosterone biosynthesis.

### 2.2 Long‐chain polyunsaturated fatty acids

The testis is rich in long‐chain polyunsaturated fatty acids (LC‐PUFA), which is primarily produced in the ER of Leydig‐ and Sertoli cells. LC‐PUFA contributes to cell membrane fluidity and plays important roles in testosterone production, and sperm function.^43^, ^44^ Elongases and desaturases such as stearoyl‐CoA desaturase‐1 (SCD1), stearoyl‐CoA desaturase‐2 (SCD2), Δ5‐ (FADS1), and Δ6‐desaturase (FADS2) modify essential fatty acids like linoleic acid and alpha‐linolenic acid.^43^, ^45^ Notably, the desaturases are di‐iron proteins. An epidemiological study found that low serum iron levels were positively correlated with decreased desaturase.

### 2.3 Spermatogenesis

It starts with a mitotic expansion of the spermatogonia. In humans, undifferentiated spermatogonia undergo one division, while differentiated spermatogonia pass through three cell divisions before entering meiosis.^46^ Particularly the relatively rapid divisions of differentiated spermatogonia pose a nutritional challenge, requiring an appropriate supply of essential nutrients, including iron, which is essential for many enzymes involved in DNA replication, proofreading, repair, and chromatin organization (Figure 3).

**FIGURE 3:**
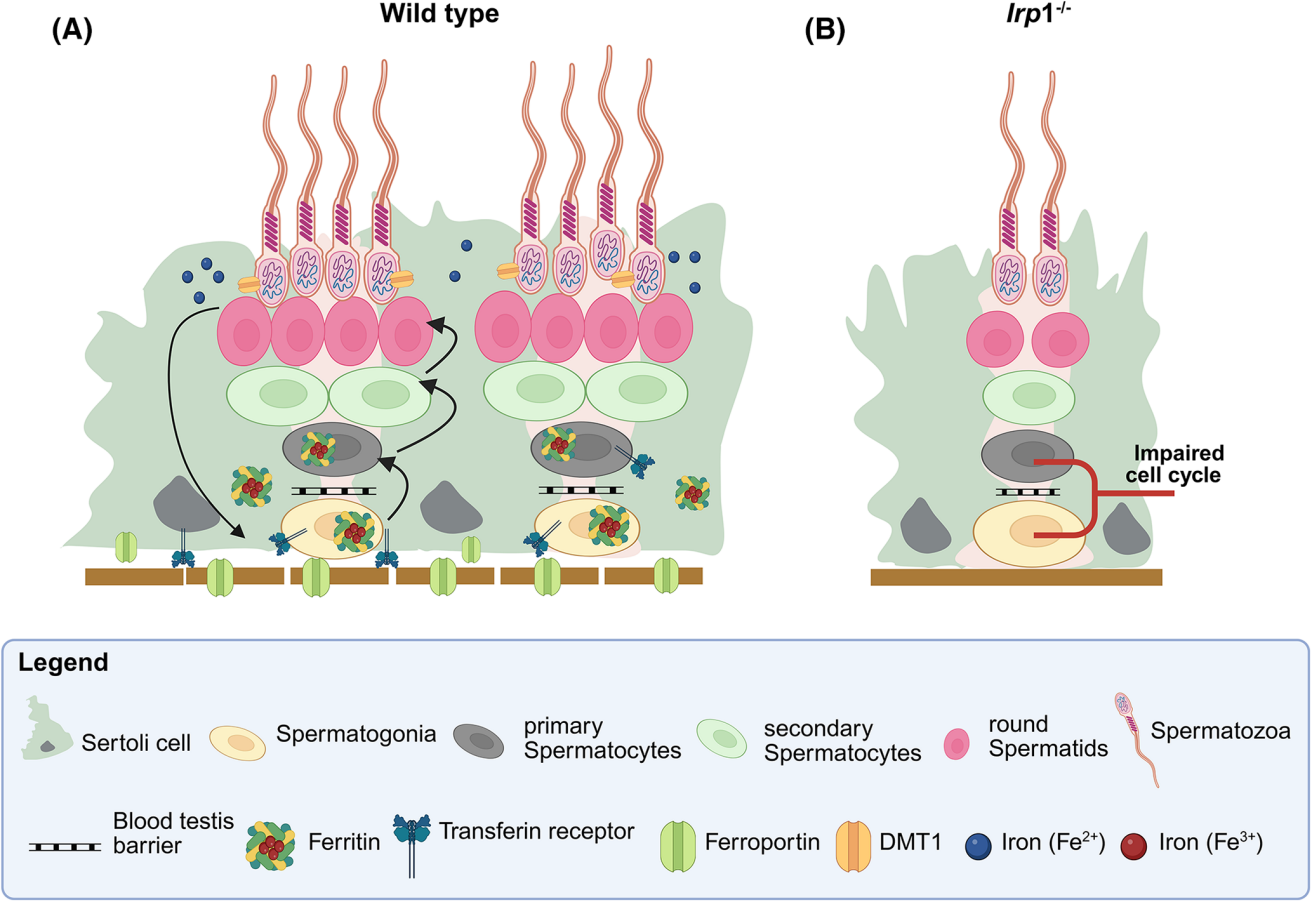
Model of iron transport and circulation in the testis. (A) This diagram illustrates iron transport within the seminiferous tubules. Spermatogonia and primary spermatocytes primarily acquire iron‐loaded ferritin. As maturation progresses, elongated spermatids return iron to the Sertoli cells (SC), which then recycle it in the form of ferritin for uptake by a new generation of germ cells. The loss of iron through released spermatozoa is compensated by iron‐bound transferrin circulating in the bloodstream. Iron uptake occurs via the transferrin receptor, while ferroportin likely plays a key role in iron trafficking across the interstitial space. (B) In the Irp1 knockout mouse model, iron transport within the seminiferous tubules remains largely unaffected; however, hypospermatogenesis is observed. This condition is characterized by a reduced number of germ cells within the seminiferous tubules. The primary cause of hypospermatogenesis in Irp1^−/−^ testis most likely is constituted by an impaired cell cycle^54^ (created with biorender.com/t2qwiqm).

In addition, large numbers of mitochondria are produced during the meiotic process, containing many heme‐ and Fe‐S cluster proteins in the respiratory complexes of the oxidative phosphorylation chain. Mitochondrial function is particularly important for meiosis.^47^ At later stages of spermiogenesis, during elongation and maturation of spermatids, many of the mitochondria and much of the cytosol are expelled through residual bodies. With the uptake of residual bodies by Sertoli cells some of the mitochondrial iron is maintained within the seminiferous tubules and it is suggested that this iron may be recycled for new rounds of spermatogenesis.

Iron homeostasis in the testis is maintained by two systems. First, iron import from the bloodstream to replace the iron lost when mature spermatids are released from the testis. Second, by an internal iron cycle consisting of iron‐containing proteins that are released from maturing spermatids during spermiogenesis, subsequently taken up by Sertoli cells which likely release their iron for re‐use.

### 2.4 Iron import from the periphery

The question of how the iron needs are met in the early steps of spermatogenesis was elegantly addressed by Michael Griswold and co‐workers in the 1980s who were the first to map the location of TFR1 in the testis.^48^ In their study of iron import from the bloodstream, they tracked the path of radioactive iron entering the seminiferous tubules. Rats were intratesticularly injected with I^125^, Fe^55^ or Fe^59^ labeled human TF. Using a time course, they demonstrated that the TF‐bound iron was likely taken up by TFR1 located on the basal membrane of Sertoli cells and different developing spermatozoa cells. Later in development, they detected radioactive iron bound to TF that was not produced by the liver, but rather locally by Sertoli cells and secreted toward the developing germ cells on both sides of the blood‐testis barrier (BTB).^49^ In addition, dietary iron could rescue the poor spermatogenesis in iron‐deficient rats,^50^ implying that iron import to the seminiferous tubules is possible. The seminiferous tubules basal membrane efficiently reduces iron influx into the seminiferous tubules in systemic iron overload,^51^ protecting the developing spermatozoa from iron overload and oxidative stress. Taken together, these studies suggested a directional flow of iron from the interstitium, where nutrients from the blood are delivered through the local capillaries, into the seminiferous tubules, and across the BTB through Sertoli cells.

### 2.5 Internal iron cycle

At the time of these studies, many iron transporters including DMT1 and FPN were not yet identified (Figure 3). Many years later, based on the subsequent distinct localization of these iron transporters and of ferritin in the seminiferous tubules, we suggested that iron may be recycled within the seminiferous tubules.^51^ Our theory was based on the specific location of DMT1 in the apical cytosol of the highly polarized Sertoli cells, an area where residual bodies are phagocytosed and their contents degraded. This made it plausible that iron is released from the iron‐rich, phagocytosed mitochondria and other cellular residues. We further suggested that this cytosolic labile iron (LIP) in the Sertoli cell cytosol is taken up by ferritin, which may then be shuttled back to the region close to the basal membrane of the Sertoli cells. In this region, where Sertoli cells surround spermatogonia, we observed that ferritin accumulates within the basal part of Sertoli cells in close vicinity of spermatogonia.

Regulation of iron homeostasis in the testis: Maintaining tightly regulated iron homeostasis in the testis is crucial to protect the developing germ cells from oxidative stress. Several regulatory systems (see above), including IRP1 and IRP2, hepcidin, NRF2, and HIF are all present in the testis.^24^, ^41^, ^52^ However, few studies have specifically analyzed their roles in maintaining testicular iron homeostasis. The BTB protects the adluminal part of the seminiferous tubules from systemic iron overload but does not inhibit iron accumulation in the basal part and the interstitium, leaving Leydig cells and testosterone production exposed to peripheral nutrient fluctuation.^51^ The IRE/IRP system, which regulates many iron‐related genes, clearly regulates ferritin expression in the testis, as ferritin is significantly overexpressed in the Sertoli cells of *Irp*2^−/−^ mice.^53^ Additionally, targeted deletion of IRP1 is linked to impaired germ cell development, although it does not ultimately result in male infertility.^54^ In the testis of *Irp*1^−/−^ mice, the expression of genes related to cell cycle regulation, DNA repair, and oxidative stress responses is reduced, suggesting that IRP1 plays an essential role in the proper development and maturation of germ cells in the testis.

Because HIF regulation is both oxygen‐ and iron‐dependent, hypoxia or iron deficiency can each lead to increased HIF activity and stabilization of the nuclear HIF complex. The HIF1α/HIFß complex has been shown to inhibit steroidogenic acute regulatory protein (STAR), which acts upstream from testosterone production in the testis, thereby reducing testosterone production.^55^ In contrast, stabilization of HIF2α has been shown to be required for normal spermatogonia function, even at normal oxygen conditions.^56^ Recently, it was reported that a 5′ RNA IRE on the HIF2α transcript recognizes IRP1 with much higher affinity than IRP2.^20^ This suggests that IRP1, which is sensitive to ROS/RNS could impair spermatogenesis when ROS/RNS levels are elevated. In addition, NRF2 plays an important role in protecting the testis from ROS/RNS‐induced damage.^57^, ^58^

## 3 FERROPTOSIS IN TESTICULAR FUNCTION AND DISEASE

Ferroptosis, a form of cell death is dependent on intracellular iron availability, and was first identified in 2012.^59^, ^60^, ^61^ Dixon demonstrated that iron accumulation within cells triggers ferroptosis, which can be inhibited by the addition of the iron chelator deferoxamine. Currently, it is understood that three key processes influence a cell's sensitivity to ferroptosis: (1) the depletion of the antioxidant glutathione (GSH) and the loss of glutathione peroxidase 4 (GPX4), (2) an increase in free iron levels, which generates ROS via the Fenton reaction, and (3) and the accumulation of lipid peroxidation products, such as 4‐hydroxynonenal and malondialdehyde, leading to cell membrane damage.^60^ Overall ferroptosis is a lipid peroxidation‐driven process. Furthermore, ferroptosis is genetically, morphologically, and biochemically distinct from apoptosis, necrosis, and necroptosis.^59^, ^60^, ^61^, ^62^ Recent research has revealed that ferroptosis can occur alongside other forms of cell death, providing also increased evidence of its potential role in testicular diseases. In numerous models of testicular injury, both apoptosis and ferroptosis have been observed to co‐exist. Notably, only the regulation of ROS production has shown the ability to inhibit both ferroptosis and apoptosis.^63^, ^64^ Iron availability, as mentioned earlier, plays a critical role in the induction of ferroptosis. This has brought autophagy and ferritinophagy into focus. On one hand, ferritinophagy has been shown to induce ferroptosis, while on the other hand, an increase in free iron through NCOA4‐mediated ferritin degradation has been demonstrated to promote ferroptosis as well.^65^, ^66^, ^67^ Consequently, ferroptosis is tightly regulated, with several pathways described in the literature to date.^68^, ^69^, ^70^

### 3.1 Mitochondrial dysfunction

Mitochondria are the primary energy source for cells and a major producer of ROS (Figure 4). In the testis, mitochondria play a critical role in spermatogenesis, especially meiosis, regulate apoptosis, which is part of normal spermatogenesis, and promote sperm motility. Previous studies have established a link between mitochondrial dysfunction and male infertility. Recently, Radaelli et al.^71^ demonstrated that mitochondrial dysfunction triggers ferroptosis, resulting in spermatogenesis‐arrest in mice (Figure 4). Another study in *Drosophila* highlighted the importance of mitochondrial ferritin in spermatogenesis, showing its protecting role against oxidative stress induced by the Fenton reaction.^72^ Furthermore, research has revealed that inhibiting ferroptosis through knockout of the mitochondrial isoform of GPX4 led to infertility in mice as evidenced by compromised sperm quality and structural abnormalities in the midpiece of the spermatozoa.^73^

**FIGURE 4:**
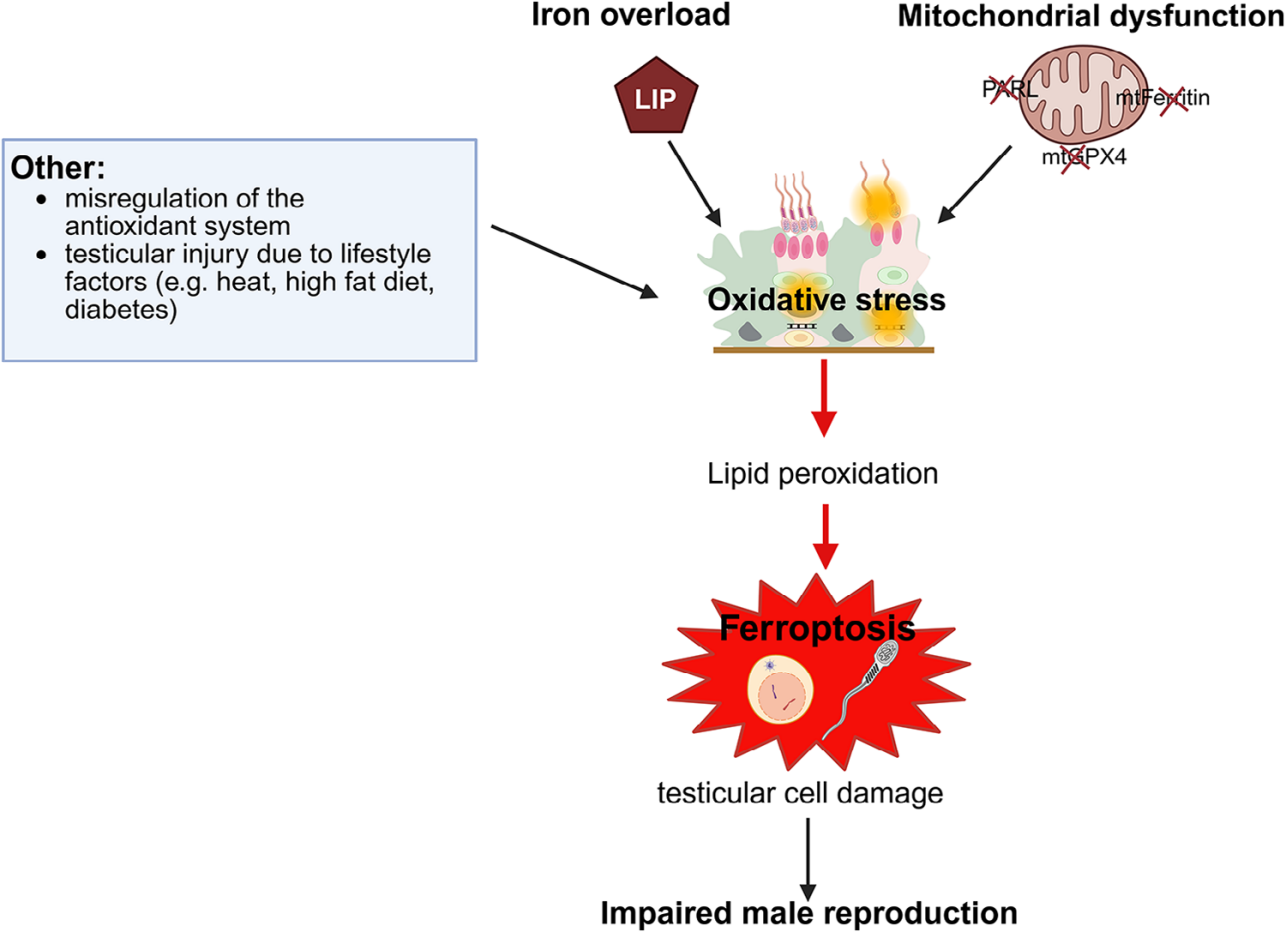
Role of ferroptosis in testicular disorders. Multiple factors contribute to the induction of ferroptosis in the testis. Recent studies have demonstrated that iron overload, particularly an increase in the labile iron pool (LIP) or mitochondrial dysfunction in specific cells, triggers high levels of oxidative stress. This, in turn, leads to lipid peroxidation (LPO), ferroptosis of mainly pachytene spermatocytes and round spermatids, and ultimately impaired male reproductive function. In addition, disruptions in the antioxidant system, along with lifestyle factors, also play a significant role in promoting ferroptosis. Testicular cell damage includes also Leydig cells as iron overload results in decreased levels of androgens (created with biorender.com/t2qwiqm).

### 3.2 Lipid peroxidation and glutathione metabolism

Increased lipid peroxidation, particularly of PUFA‐containing phospholipids in cell membranes, serves as a critical marker of ferroptosis (Figure 4). Spermatozoa are particularly vulnerable to free radical damage because of their LC‐PUFA‐rich membranes and lack of cytoplasmic ROS scavengers.^74^ Protection from ROS is typically regulated by glutathione peroxidase 4 (GPX4) and the antiporter SLC7A11. SLC7A11, predominantly expressed in Sertoli and Leydig cells, plays a vital role in maintaining the cysteine/GSH redox balance, which is essential for protecting germ cells from oxidative stress.^75^, ^76^ Studies have shown that SLC7A11 knockout mice exhibit reduced reproductive capacity, characterized by an increased proportion of immature spermatozoa. Additionally, diminished SLC7A11 and GPX4 expression have been associated with testicular injury and infertility.^70^, ^75^, ^77^, ^78^ These findings underscore the importance of a functional antioxidant system for preserving male reproductive health.

### 3.3 Testicular injury and disease

As noted, only a limited number of studies have investigated the relationship between testicular diseases and ferroptosis (Figure 4). Much of the existing research focuses on environmental pollutants, such as the increased exposure to plastic substances, and their impact on fertility in both men and mice. Plastic components such as tetrabromobisphenol A, and di(2‐ethylhexyl) phthalate are well‐known to disrupt spermatogenesis. Recent studies have also linked these substances to heightened sensitivity to ferroptosis.^79^, ^80^, ^81^, ^82^ Beyond plastic components, heavy metals such as cadmium, agricultural toxicants, and chemotherapeutic agents have been shown to promote ferroptosis activation in the mouse testis. For instance, a study in mice treated with the chemotherapeutic drug busulfan reported a substantial reduction in sperm concentration and motility, culminating in azoospermia. Here, azoospermia was elicited by busulfan‐induced ferritinophagy and an imbalanced antioxidant system either as the primary cause or further downstream. Notably, these effects were partially reversed through ferroptosis inhibition using ferrostatin‐1 (a lipid ROS and ferroptosis inhibitor) as well as via deferoxamine (an iron chelator).^83^, ^84^, ^85^

Further evidence comes from studies on oligospermia patients, who exhibited reduced levels of GPX4 and NRF2 protein in their spermatozoa compared with healthy men. In a detailed investigation, Han et al.^86^ used an *Nrf*2^−/−^ mouse model to demonstrate that the deletion of *Nrf*2 impaired sperm quality and motility while inducing ferroptosis in spermatogenic cells via inhibition of the SLC7A11/GSH/GPX4 pathway. Remarkably, the administration of ferrostatin‐1 reversed these detrimental effects.

Overall, the studies conducted to date clearly demonstrate that ferroptosis plays a pivotal role in specific types of testicular injuries. A deeper understanding of the precise mechanism underlying this process could pave the way for developing new strategies to prevent detrimental impairment of spermatogenesis in the future.

## 4 TESTICULAR DISEASE AND THE ROLE OF IRON

Iron plays a critical role in spermatogenesis, circulating within the germinal epithelium to support this essential process and to minimize loss originating from the release of spermatozoa into the lumen of the seminiferous epithelium.^53^, ^87^ In the following chapter, we will explore the effects of the variation of iron concentration on spermatogenesis, along with the alterations observed during bacterial or viral infections and chronic diseases.

### 4.1 Changes in testicular function under high and low iron conditions

#### 4.1.1 Iron overload

Elevated systemic iron levels, referred to as iron overload, occur when TF saturation exceeds 45% in women and 50% in men. If serum TF saturation surpasses 60%, iron accumulation in tissues can cause significant damage.^88^ Systemic factors often underlie increased iron concentrations, such as repeated blood transfusions or excess iron intake from dietary supplements. Additionally, genetic conditions like hereditary hemochromatosis and beta‐thalassemia major are notable causes of elevated iron levels.

In the context of male reproduction, to our knowledge, no studies have directly demonstrated iron accumulation in the testis because of impaired systemic iron homeostasis. Available research has shown changes in male reproduction stemming from systemic iron overload. This points to a unique ability of the testis to maintain iron homeostasis. An autonomous iron circulation system within the seminiferous tubules may contribute to the safeguarding of spermatogenesis from fluctuations in systemic iron levels.^53^ Human studies on hereditary hemochromatosis and beta‐thalassemia major have demonstrated alterations in spermatogenesis and spermatozoa functional parameters. Hereditary hemochromatosis, often caused by mutations in the HFE gene, results in reduced levels of plasma hepcidin and subsequent increased intestinal iron absorption. While frequently asymptomatic or diagnosed late, the condition can lead to significantly elevated systemic iron levels (reviewed in a study by Hollerer et al.^89^). In male reproduction, patients with hereditary hemochromatosis exhibit reduced libido and abnormal semen parameters compared with healthy individuals, along with altered hormone levels.^90^, ^91^ Excessive iron accumulation in the testis disrupts Leydig and Sertoli cell function, contributing to these observed changes. Some studies suggest a link between hypogonadism and hereditary hemochromatosis, potentially explaining the hormonal abnormalities, although this connection remains debated in the literature.^92^, ^93^, ^94^, ^95^ Advances in medical research, including genetic testing and patient screening, have led to effective treatments, making hereditary hemochromatosis a rare cause of impaired spermatogenesis. For a far more comprehensive understanding of this condition, readers are referred to recently published reviews.^96^, ^97^, ^98^

Beta‐thalassemia major is a genetic disorder that is more prevalent in the Mediterranean region and the Maldives. It arises from point mutations in the β‐globin chain (HBB) gene, leading to defective β‐globin synthesis, premature erythrocyte destruction, and anemia. Patients typically receive life‐long blood transfusions, which result in iron overload, and the ineffective erythropoiesis additionally leads to excessive iron absorption.^99^, ^100^ The resulting severe iron overload affects organs such as the liver, heart, and reproductive system when not treated with regular chelation therapy.^101^ Iron deposition in the pituitary gland and testis disrupts the hypothalamic‐pituitary‐gonadal axis, leading to increased oxidative stress. This disruption delays puberty and alters sperm parameters in over 50% of patients.^102^, ^103^, ^104^ As a result, conditions such as oligozoospermia and asthenozoospermia are observed. Elevated serum ferritin levels reflect iron overload and are also associated with compromised sperm quality, including reduced sperm counts, abnormal morphology, and increased DNA fragmentation in some patients.^105^ Early intervention with iron chelators has shown promise in reducing iron‐related damage and supporting sexual development. However, it needs to be noted that some studies have reported an association between iron chelation therapy and hypogonadism.^103^, ^106^

#### 4.1.2 Iron deficiency

Iron deficiency is a global health issue, affecting more than 2 billion people worldwide.^107^ Across all ages, males have a lower prevalence of anemia than females, nonetheless, 17.5% of men were affected.^108^ This is of relevance as male reproduction is also influenced by inadequate iron levels. Low iron availability can impair spermatogenesis, a condition typically arising from an iron‐deficient diet or poor intestinal absorption. The latter is often associated with conditions such as inflammatory bowel disease or chronic iron loss.^109^, ^110^ IBD creates a hypoxic environment that indirectly disrupts spermatogenesis and androgen synthesis. Seminiferous tubules, being highly metabolically active, are especially sensitive to changes in oxygen tension. These alterations can result in reduced testicular volume and compromised sperm quality such as DNA damage, and decreased sperm counts, for example, by meiotic arrest, abnormal morphology, and impaired motility.^109^, ^111^, ^112^ However, studies have demonstrated that iron supplementation can effectively reverse these adverse effects, restoring both sperm quality and testosterone levels to normal.

### 4.2 Role of iron in bacterial and viral infection of the testis

#### 4.2.1 Bacterial infection

During bacterial infections, the host often restricts iron availability to pathogens as part of the immune response,^113^ which affects both the pathogen and the host. Iron plays a crucial role in regulating the immune response, including processes such as macrophage polarization.^114^, ^115^ However, certain bacterial species, such as uropathogenic *Escherichia coli* (UPEC) pathovars, have evolved mechanisms to extract iron from the host, potentially enhancing their virulence.^116^, ^117^, ^118^, ^119^, ^120^

The most common bacterial infection of the male reproductive tract is acute epididymo‐orchitis, typically caused by UPEC strains or sexually transmitted pathogens (e.g., *Chlamydia trachomatis*).^121^ Bacterially induced epididymo‐orchitis is an ascending infection from the urethra that initially affects the epididymis and, in most cases (90%), subsequently spreads to the testes.^122^, ^123^ Studies have shown that 40% of patients with UPEC‐induced epididymo‐orchitis experience subfertility or infertility, even after successful antibiotic treatment, because of a persistently reduced sperm count.^123^, ^124^, ^125^, ^126^, ^127^ Consequently, infectious diseases and inflammation of the genital tract are among the leading causes of male fertility problems.^128^ This raises the question of how alterations in testicular iron homeostasis influence the progression of UPEC‐induced epididymo‐orchitis. A recent study addressed by use of global knockout mouse models for iron regulatory proteins (IRP)1 and IRP2 in animals infected with UPEC. The findings revealed contrasting roles for the knockout models during infection. Infected *Irp2* ^−/−^ mice exhibited a heightened immune response, resulting in greater tissue damage compared with wild‐type mice. In contrast, infected *Irp1* ^−/−^ mice displayed a reduced immune response, leading to less tissue damage than wild‐type mice. This effect was attributed to diminished ERK1/2 signaling, which resulted in a decreased release of proinflammatory cytokines and, consequently, less tissue damage.^129^ Overall, this study underscores the complex interplay between iron metabolism, immune response, and tissue damage offering valuable insights into potential therapeutic approaches for managing male fertility issues related to bacterial infection.

#### 4.2.2 Viral infection

Over recent decades, several viruses have been implicated with isolated orchitis, epididymo‐orchitis, and subsequent infertility. These include sexually transmitted viruses such as HIV‐1 and Zika virus (ZIKV), as well as the mumps virus (MuV), which has long been known to be associated with these conditions.^130^, ^131^, ^132^, ^133^, ^134^ Moreover, some studies have linked SARS‐CoV‐2 to testicular tissue damage and orchitis, although the causal relationship remained unclear.^135^, ^136^, ^137^ In addition, it was shown that Fe–S clusters play a crucial role in the proper functioning of the SARS‐CoV‐2 RNA‐dependent RNA polymerase, ensuring the accurate replication of the viral genome. These studies show that Fe‐S clusters could be promising targets for COVID‐19 therapy.^138^, ^139^ Collectively, these findings highlight the significant impact of viral infections on male reproduction. As described earlier in this review, infections can disrupt iron homeostasis, and viral infections are no exception. For a more comprehensive discussion of this topic, the following excellent reviews are recommended,^140^, ^141^ as we will only briefly address it here.

To illustrate this, we present two examples of how viruses alter iron metabolism. HIV‐1, which resides in macrophages, interacts with the HFE protein through the Nef protein sequestering HFE in the trans‐Golgi apparatus. This interaction increases iron concentrations within macrophages, creating an environment conducive to viral replication but harmful to the host. Elevated iron levels are, therefore, associated with poorer disease outcomes.^142^, ^143^ In contrast, the Zika virus employs a different strategy by exploiting ferroptosis. It manipulates lipid metabolism to support its viral life cycle, thereby creating conditions that enhance its replication and persistence.^144^

As with bacterially induced epididymo‐orchitis, no studies have specifically examined the effects of viral infections on iron homeostasis in the testis. Therefore, it can only be hypothesized that the overall iron distribution in the testis remains largely unchanged. However, certain viral infections may induce localized changes, such as an increased iron concentration in macrophages or heightened ferroptosis. These alterations, combined with elevated inflammatory factors, could exacerbate tissue damage.

### 4.3 Role of iron in chronic and autoimmune testicular disease

Chronic or autoimmune diseases are typically characterized by heightened inflammation. While no studies to date have specifically examined the relationship between autoimmune orchitis and iron homeostasis, it is reasonable to hypothesize that iron metabolism may be affected. Research has shown that immune cells, such as T cells and macrophages in experimental autoimmune orchitis (EAO), secrete various pro‐inflammatory cytokines, including TNF‐α and IL‐6.^145^, ^146^, ^147^ IL‐6 and in certain cases, TNF‐α (e.g., in adipose tissue) are known to regulate the expression of hepcidin in the liver during inflammatory reactions.^148^, ^149^ In the context of EAO, hepcidin inhibition of ferroportin could lead to iron accumulation, primarily in macrophages, potentially contributing to tissue damage through the generation of ROS. However, these hypotheses, remain theoretical and require validation through dedicated studies.

Diabetes and obesity are two chronic diseases that are becoming increasingly prevalent in industrialized nations. Currently, over 500 million people worldwide are affected by diabetes, and this number is expected to rise in the coming years.^150^ A decline in male fertility has already been documented, with studies reporting reduced sperm counts and motility in these patients.^151^, ^152^, ^153^, ^154^

One potential explanation for these observations is the altered iron metabolism observed in obese individuals, who are more susceptible to iron deficiency. Hepcidin plays a key role in this process.^155^, ^156^, ^157^ Additionally, a clinical study has reported increased levels of IL‐6 and hepcidin in sperm, linking these findings to microRNAs associated with inflammation and iron metabolism.^158^ However, the relationship between these changes and testicular iron homeostasis remains unclear and warrants further investigation (Table 1).

**TABLE 1:** Summary of diseases with influence on iron homeostasis and effect on male sexual functions.

	Disease	Reproduction dysfunction	Literature
Iron overload	Hereditary hemochromatosis (mutation in HFE gene)	Decreased libido Atypical semen parameters Altered hormone levels	^87^, ^88^, ^89^
	Beta‐thalassemia	Delay in puberty Altered sperm parameters	^100^, ^101^, ^102^, ^103^, ^104^
Iron deficiency	Inflammatory bowel	Affects testicular volume Reduced sperm quality	^107^, ^109^, ^110^
	Diabetes/Obesity	Reduced testosterone levels Low sperm motility Altered sperm morphology	^153^, ^154^, ^155^
Bacterial/viral infection	Epididymo‐orchitis	Sub‐ or infertility (persistently reduced sperm counts)	^119^, ^120^, ^121^, ^122^, ^123^, ^124^, ^125^, ^126^

## 5 CONCLUSION AND OUTLOOK

Iron is indispensable for male reproductive health, playing a crucial role in testosterone synthesis, spermatogenesis, and testicular cell function. The delicate balance of iron homeostasis is maintained through sophisticated systemic and cellular regulatory mechanisms, ensuring an adequate iron supply while preventing toxic overload. However, disruptions in iron metabolism—whether through genetic mutations, nutritional deficiencies, infections, or chronic diseases—can have profound consequences on male fertility. Targeting iron homeostasis through pharmacological and dietary interventions could prevent iron‐related infertility. Particularly, ferroptosis inhibitors offer potential therapeutic strategies to mitigate oxidative stress‐induced testicular damage, while iron‐based biomarkers could serve as diagnostic tools for assessing male fertility risks. In summary, iron regulation in the testis is critical for reproductive health, and dysregulation can lead to infertility. Future studies should focus on therapeutic approaches to restore iron balance and prevent testicular dysfunction, including those caused by microbial infection of the male reproductive tract.
